# Extraction of saponins and toxicological profile of *Teucrium stocksianum* boiss extracts collected from District Swat, Pakistan

**DOI:** 10.1186/0717-6287-47-65

**Published:** 2014-12-04

**Authors:** Syed Muhammad Mukarram Shah, Abdul Sadiq, Syed Muhammad Hassan Shah, Shahzeb Khan

**Affiliations:** Department of Pharmacy, University of Malakand, Chakdara, Dir, Pakistan; Department of Pharmacy, Sarhad University of Science and Information Technology, Peshawar, KPK, Pakistan

**Keywords:** Saponins, *Teucrium stocksianum*, Cytotoxicity, Phytotoxicity

## Abstract

**Background:**

The current era is facing challenges in the management of neoplasia and weeds control. The currently available anti-cancer and herbicidal drugs are associated with some serious side effects. Therefore numerous researchers are trying to discover and develop plant based alternative particularly for the rational management of cancer and weed control. *Teucrium stocksianum* possess antioxidant and analgesic activities. The current study was designed to evaluate crude saponins (CS), methanolic extract and sub-fractions of *T. stocksianum* for cytotoxic and phytotoxic potentials. CS, methanolic extract and sub-fractions were extracted from powdered plant material using different solvents. Cytotoxic potential of the extracts at a dose of 10, 100 and 1000 μg/ml were evaluated against Brine shrimp’s nauplii. Phytotoxic assay also performed at the same concentration against *Lemna minor*. Etoposide and Paraquat were used as positive controls in cytotoxic and phytotoxic assays respectively.

**Results:**

The percent yield of crude saponins was (5%). CS demonstrated tremendous brine shrimp lethality showing < 10 μg/ml LC_50_. The *n*-hexane (HF) and chloroform fractions (CF) demonstrated excellent cytotoxicity with 80 and 55 μg/ml LC_50_ respectively. Whereas the methanolic extract (TSME), ethyl acetate (EAF) and aqueous fractions (AF) revealed moderate cytotoxicity showing 620, 860 and 1000 μg/ml LC_50_ values respectively. In phytotoxic assay profound inhibition was displayed by HF (96.67%) and TSME (95.56%, 30 μg/ml LC_50_) against the growth of *Lemna minor* at 1000 μg/ml respectively. Both CF and EAF demonstrated profound phytoxicity (93.33%) respectively at highest concentration (1000 μg/ml), while AF and CS demonstrated weak phytotoxicity with 1350 and 710 μg/ml LC_50_ values respectively.

**Conclusion:**

Cytotoxicity and phytotoxicity assays indicated that the crude saponins, *n*-hexane and chloroform fractions of *T. stocksianum* could play a vital role in the treatment of neoplasia and as potential natural herbicides. Therefore these sub-fractions are recommended for further investigation with the aim to isolate novel anti-cancer and herbicidal compounds.

## Background

Plants are the natural source of a number of drug molecules [1]. Maximum population of the world depends upon the natural medicines formulated from plant sources. Researchers are trying to explore various types of plants for their potential tendency to treat various challenging diseases. The most prominent disease which demands an urgent attention and rational investigation is neoplasia [2]. In addition to the medicinal use of plants, these are one of the major sources of income of utmost world population. To increase the production of crops different strategies are employed, one of the strategies is the use of weed killers [3]. The synthetic herbicidal agents are very much effective to control weeds in variety of crops. Nonetheless, most of them are associated with cumbersome adverse effects [4]. As far as the plant sources are concerned, they are void of these unwanted effects. A number of phytochemicals are famous for certain biological and chemical activities for instance, most of the alkaloids are responsible for antimicrobial activity [5], flavonoids and phenols possessing antioxidant activity [6, 7]. Saponins are group of compounds which have a number of pharmacological activities including anti-inflammatory, antioxidant, anticancer, insecticidal, anthelmintic and antimicrobial [8–12].

A range of isolated compounds from plant material contributed a lot in the field of clinical research and therapy [13]. The plants having allelopathic activity helped in the development of safe and potent herbicidal agents which increased the per acre production in agricultural sector [14].

*Teucrium stocksianum* (Lamiaceae) and various species of the genus *Teucrium* are ethnomedicinally used as analgesic, antipyretic, anticancer and anthelmintic [15–18]. *T. royleanum* Wall.ex.Benth (Lamiaceae) possesses strong antimicrobial activity and excellent inhibitor of Acetylcholine esterase and Butyrylcholine esterase [19, 20]. *T. stocksianum* is used ethnomedicinally as expectorant, antipyretic, anticancer, anti-malarial, bitter and sore throat [21–23]. The essential oils of *T. stocksianum* has experimentally been reported as a strong analgesic [24]. Based on the thorough review of the available literature on the folk uses of this plant and the extreme necessity for safe, efficacious and economic anticancer and herbicidal drugs, the present study was designed to evaluate the crude saponins and various other samples of *T. stocksianum* for cytotoxic and phytotoxic activity.

## Results

### Crude saponins

Powdered plant material (20 g) of *T. stocksianum* yielded about 1 g (5%) of crude saponins.

### Brine shrimp cytotoxicity effect

The cytotoxic potential of CS of *T. stocksianum*, methanolic extract (TSME) and its sub-fractions against brain shrimps were evaluated at different concentrations as shown in Table 1. CS at a test concentration of 10, 100 and 1000 μg/ml caused, 55.55, 63.33 and 94.44%, cytotoxicity respectively which is prominent compared to the other extracts with LC_50_ < 10 μg/ml (Table 1). TSME demonstrated 58.88, 35.55 and 18.88% cytotoxicity at concentrations of 1000, 100 and 10 μg/ml with LC_50_ 620 μg/ml. The *n*-hexane fraction has shown a practical cytotoxic effect (67%) at 1000 μg/ml, and moderate at 100 μg/ml (42%) and 10 μg/ml (38%) lethality with LC_50_ of 80 μg/ml. Chloroform caused 71, 60 and 42% lethality with LC_50_ of 55 μg/ml, while the ethyl acetate fraction displayed 54, 28 and 16 lethal effect with LC_50_ 860 μg/ml (Table 1). The aqueous fraction also displayed reasonable cytotoxic potential at all test doses, i.e. 49, 27 and 12% with LC_50_ 1000 μg/ml. Etoposide was used as standard drug (LC_50_ 9.8 μg/ml).

**Table 1:** **Concentration dependent cytotoxic potential of crude methanolic extracts of** ***T. stocksianum*** **and its sub-fractions against Brine shrimps nauplii**

Samples/Drug	Conc 10 μg/ml	Conc 100 μ/ml	Conc 1000 μ/ml	LC _50_μ/ml
**Crude saponins**	55.555 ± 1.112%	63.332 ± 3.337%	94.444 ± 2.943	< 10
**Methanolic extract**	18.888 ± 5.886%	35.555 ± 2.943%	58.887 ± 2.944%	620
***n-*** **hexane fraction**	37.777 ± 2.943%	42.221 ± 2.943%	67.777 ± 2.224%	80
**Chloroform fraction**	42.221 ± 2.943%	59.999 ± 1.926%	71.110 ± 2.943%	55
**Ethyl acetate fraction**	16.666 ± 1.926%	28.888 ± 1.112%	54.444 ± 2.943%	860
**Aqueous fraction**	12.221 ± 2.943%	27.777 ± 2.943%	49.998 ± 1.926%	1000

Conc; concentration. Values are expressed as the mean ± SEM of three independent observations. Standard drug; Etoposide LD_50_ = 9.8 μg/ml.

### Phytotoxic effect

The HF fraction has shown maximum phytotoxic effect as compared to other tested samples of *T. stocksianum* against *Lemna minor* as shown in Table 2. HF has shown remarkable potential, 96.67 ± 1.90, 82.23 ± 2.90 and 51.10 ± 1.10% at a concentration of 1000, 100 and 10 μg/ml respectively, with LC_50_ of 9.8 μg/ml. TSME at lowest concentration (10 μg/ml) displayed good effect (27.76 ± 1.90 at 10 μg/ml), excellent activity (71.10 ± 1.10% at 100 μg/ml) and outstanding potential (95.56 ± 1.10 at 1000 μg/ml), as shown in Table 2. Both CF and EAF have shown significant effect (93.33 ± 1.90 and 93.33 ± 1.10) respectively at highest concentration (1000 μg/ml). At a 100 μg/ml CF and EAF have shown 56.66 ± 1.90 and 63.33 ± 1.10% growth inhibitions respectively, while both AF and CS demonstrated weak phytotoxic potential, with LC_50_ 1350 and 710 μg/ml respectively. Moreover, CF was more potent at lowest concentration (10 μg/ml) as compared to EAF.

**Table 2:** **Concentration dependent phytotoxic potential of crude methanolic extracts of** ***T. stocksianum*** **and its sub-fractions against** ***Lamna minor***

Sample	Conc; 1000 μg/ml	Conc; 100 μg/ml	Conc; 10 μg/ml	LC _50_μg/ml
**Crude saponins**	32.99 ± 2.502%	19.99 ± 1.925%	7.77 ± 1.113%	1350
**Crude methanolic extract**	95.56 ± 1.10%	71.10 ± 1.10%	27.76 ± 1.90%	30
***n*** **-hexane fraction**	96.67 ± 1.90%	82.23 ± 2.90%	51.10 ± 1.10%	9.8
**Chloroform fraction**	93.33 ± 1.90%	56.66 ± 1.90%	42.23 ± 2.90%	50
**Ethyl acetate fraction**	93.33 ± 1.10%	63.33 ± 1.10%	33.33 ± 1.90%	48
**Aqueous fraction**	55.55 ± 2.223%	37.77 ± 1.113%	18.88 ± 1.113%	710

Conc; concentration. Values are expressed as the mean ± SEM of three independent observations. Standard drug; Paraquat, LD_50_ = 0.90 μg/ml.

## Discussions

Evaluation of plants extract candidly helped in the exploration and isolation of numerous precious compounds which aid a lot in the prevention and treatment of various ailments. As far as the cancer therapy is concerned, a large number of plants are scientifically validated by investigators to cope with this disease [25, 26]. Valuable compounds, especially the saponins have also been isolated and purified from many plants for cancer therapy [27]. The cytotoxic activity performed against brine shrimps demonstrated that *T. stocksianum* contains strong anticancer activity. The saponins showed awesome cytotoxic effect (LC_50_ < 10 μg/ml) in this study. The cytotoxic effect of saponins has previously been reported by a number of researchers [8]. Saponins are group of secondary metabolites which have a plethora of pharmacological activities, like anthelmintic [11], insecticidal [10] and antioxidant [9]. The anticancer potential of saponins present in *T. stocksianum* is therefore verified in this activity against brine shrimp, as there is a positive correlation between human nasopharyngeal carcinoma and brine shrimp lethality [17]. The CF (LC_50_ < 55 μg/ml) and HF (LC_50_ < 80 μg/ml) also revealed high cytotoxic activity which may be due to the presence of high quantity of saponins and other cytotoxic compounds in these fractions.

Various herbicidal agents used against weeds in agricultural crops resulted in numerous adverse effects. To control the adverse effects caused by the synthetic herbicides, a range of natural herbicides have been isolated from plant sources [28]. In the present study the phytotoxicity assay divulged that *T. stocksianum* is having strong phytotoxic activity and from the Table 2 it can be concluded that the activity was dose dependent i.e. the phytotoxic activity increased by increasing the dose of extract. The highest phytotoxic activity, shown by TSME (95%) and HF (96%) fraction at the concentration of 1000 μg/ml, disclosed that these plant samples are rich in active moieties, responsible for the phytotoxic action. Table 2 revealed that the plant extracts of *T. stocksianum* could be an excellent source of natural herbicides. Based on the present literature and experimental verification it is worthwhile to be noticed that potentially *T. stocksianum* may be a best source of economical and efficient anticancer and natural herbicides. Owing to this probable paramount activity the different fractions rich in active principles and crude saponins should be further subjected to bioassay guided isolation in ordered to get novel, effective, economical and safe moieties for the rational management of carcinoma and weeds eradication.

## Conclusion

Preliminary phytochemical screening indicated the presence of saponins along with other secondary metabolites. The crude saponins, the methanolic extract and resultant subfractions have demonstrated outstanding cytotoxic activity. Furthermore the methanolic extract and resultant subfractions have also displayed remarkable potential except CS and AF, in phytotoxicity assay. On the basis of our results we could aspect that the extracts, particularly the crude saponins could play a major role in the treatment of the current challenging disease like cancer and the n-hexane subfraction of *T. stocksianum* as herbicide. Therefore CS and HF need further scientific investigation to isolate bioactive compounds for the rational and effective use as an anticancer and natural herbicide.

## Methods

### Plant material

*T. stocksianum* was collected in the month of August 2012, from Marghuzar valley of District Swat, in the province of Khyber Pakhtunkhwa (KPK), Pakistan. Plant was identified by Professor Dr. Nasrullah Department of Botany University of Malakand Chakdara Dir, Khyber Pakhtunkhwa, Pakistan. A voucher specimen (H.UOM.BG.199b) has been deposited in the Herbarium of the same Department for future reference.

### Chemicals

Methanol, *n*-hexane, chloroform, ethyl acetate (analytical grad) were purchased from Merck, Pakistan. While Paraquat and Etoposide both were procured from Sigma Aldrich having 98% purity.

### Extraction

Plant was cleared, washed with tape water to remove dirt and was cut into small pieces. It was dried in shade, pulverized to coarse powder and was stored in polyethylene bags for future use. About 500 g was soaked for one week in methanol (80%). The methanolic extract was passed through nylon cloth and then filtered through Whattman’s No 1 filter paper. The extract was concentrated under reduced pressure using rotary evaporator at controlled temperature (40–45°C). The percent yield of the extract was 6.2% (31 g) [29].

### Fractionation

The methanolic extract (31 g) was fractionated with different organic solvents. The extract was transferred to 1.0 liter separating funnel and was suspended in about 500 ml distilled water. The aqueous extract was partitioned with an equal volume (500 ml) of *n*-hexane, chloroform and ethyl acetate which yielded about 4 g *n-*hexane, 6 g chloroform, 5 g ethyl acetate and 12 g of aqueous fractions respectively [30].

### Extraction of crude saponins

Crude saponins (CS) from *T. stocksianum* were extracted by heating the powdered sample (20 g) for 4 h at 55°C with 100 ml of ethanol (20%). The extract was filtered and residue was re-extracted with 200 ml of ethanol (20%). The extract was concentrated on water bath till the volume reduced to 40 ml, which was mixed with 20 ml diethyl ether in a separating funnel. The mixture was vigorously shacked and then the separating funnel was fixed in a stand till the development of aqueous and diethyl layer. Aqueous portion was collected while the diethyl ether portion was discarded. To the aqueous layer *n*-butanol (60 ml) was added and properly mixed by vigorous shaking. The *n*-butanol extract was treated with 10 ml of 5% NaCl solution. The resultant solution was concentrated on a water bath and the cured saponins were dried in an oven [31].

### Brine shrimp lethality bioassay

Stock solution of crude saponins of *T. stocksianum*, methanolic extract and its subsequent fractions were prepared by dissolving 20 mg of the extracts in 2 ml of methanol. Then 10, 100, and 1000 ppm concentrations were prepared by taking 5, 50, and 500 μl respectively from the stock solution in three separate vials. The solvent (methanol) from each vial was evaporated at room temperature and 10 shrimp’s nauplii were transferred to each vial with the help of dropper. In each vial 5 ml of simulated sea water (sea salt 38 g/l of distilled water, pH 7.4) was added. The negative control vials contained 10 shrimps and 5 ml of sea water. Each dilution was prepared in triplicate. All the vials were incubated at 25–27°C under illumination and the survivor were counted after 24 h [32]. Etoposied was used as a positive control. Percent mortality was determined using the following formula;


### Phytotoxic activity

In-vitro phytotoxic activity of the various dilutions of crude saponins of *T. stocksianum*, methanolic extract and its sub-fractions were conducted against *Lemna minor.* Media was prepared by dissolving various inorganic components in 100 ml of distilled water and pH was adjusted to 6.0-7.0 by adding KOH solution. Media was sterilized at 121°C, 15 psi for 15 min in autoclave. Stock solution was prepared by dissolving 10 mg of the extract in 1 ml of ethanol. Three sets of three different concentrations (10, 100 and 1000 μl) were prepared in flasks from the stock solution. The solvent was evaporated from the flask in aseptic environment. To each flask 20 ml of the pre-sterilized medium and 10 plants each having a rosette of three fronds, was added. Two other flasks were prepared containing solvent and standard phytotoxic drug (Paraquat), served as negative and positive control respectively. Afterwards, the flasks were properly plugged with cotton and were transferred to growth cabinet for seven days. On the 7th day each flask was observed and the number of fronds was counted. The percent growth inhibition was analyzed with reference to the negative control [33].
